# Supplementary material for: Preclinical Evidence of Progesterone as a New Pharmacological Strategy in Human Adrenocortical Carcinoma Cell Lines
Source: Int J Mol Sci. 2023 Apr 6;24(7):6829. doi: 10.3390/ijms24076829 (PMC10095539; doi:10.3390/ijms24076829)
Supplement: Supplementary file 1 [file ijms-24-06829-s001.zip › ijms-2277611-supplementary.pdf]

Pg toxicity on wild-type (AB) strain zebrafish embryos.

Preliminary experiments were conducted to evaluate the Pg toxicity on wild-type (AB) strain zebrafish embryos. Doses higher than 25 µM caused 100% mortality. Exposure to 25 µM Pg was not lethal, but nearly 50% of embryos developed pericardial edema and yolk sac edema (Supplementary Figure 5) while 10 µM Pg was a safe dose.

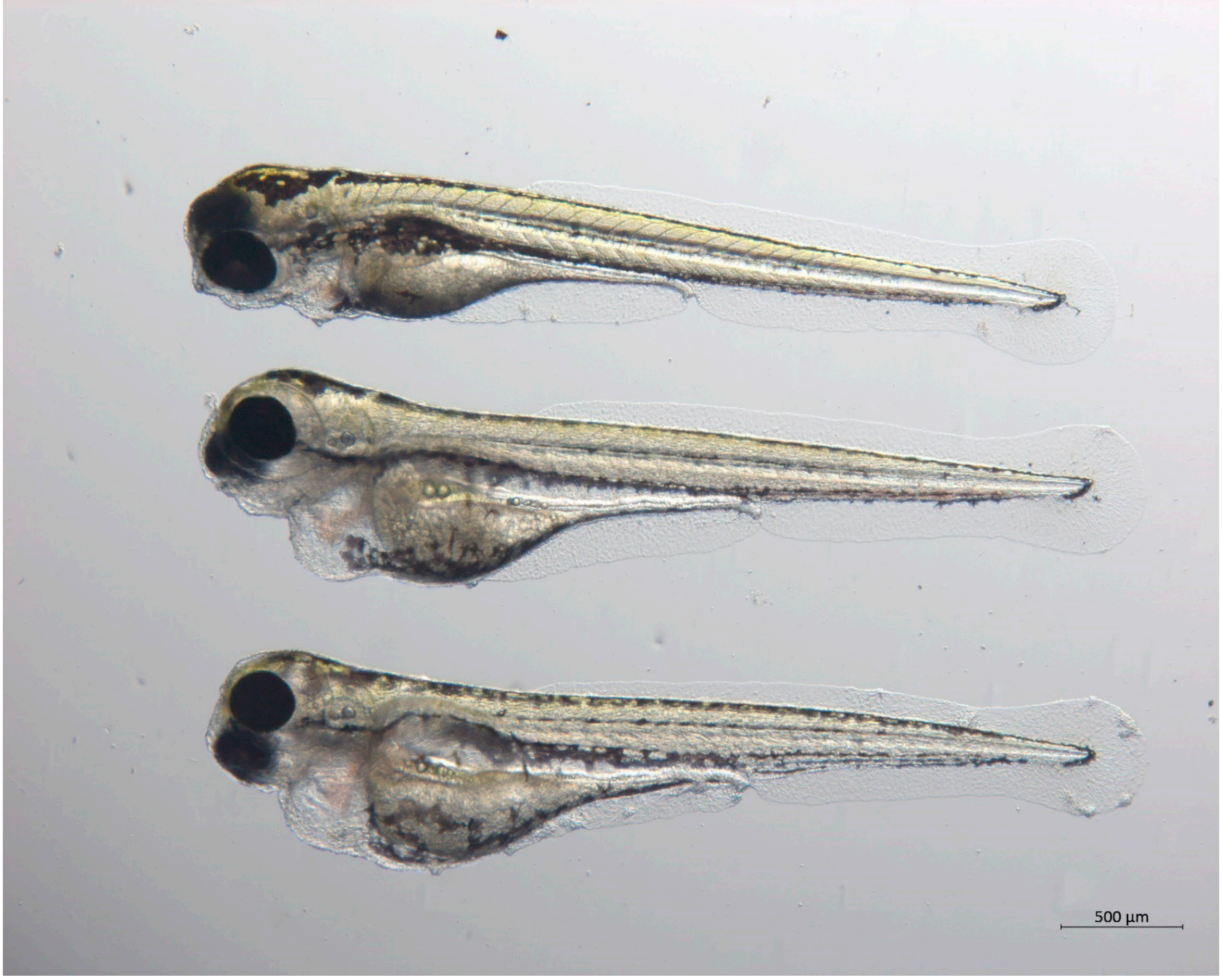

Supplementary Figure S1. Representative, lateral-view, picture of 120 hpf wild-type (AB) strain zebrafish embryos treated with 25 µM of Pg. Different entities of pericardial edema and yolk sac edema were shown. A picture has been acquired using a Zeiss Axiozoom V13 microscope with white light at 10x magnification.

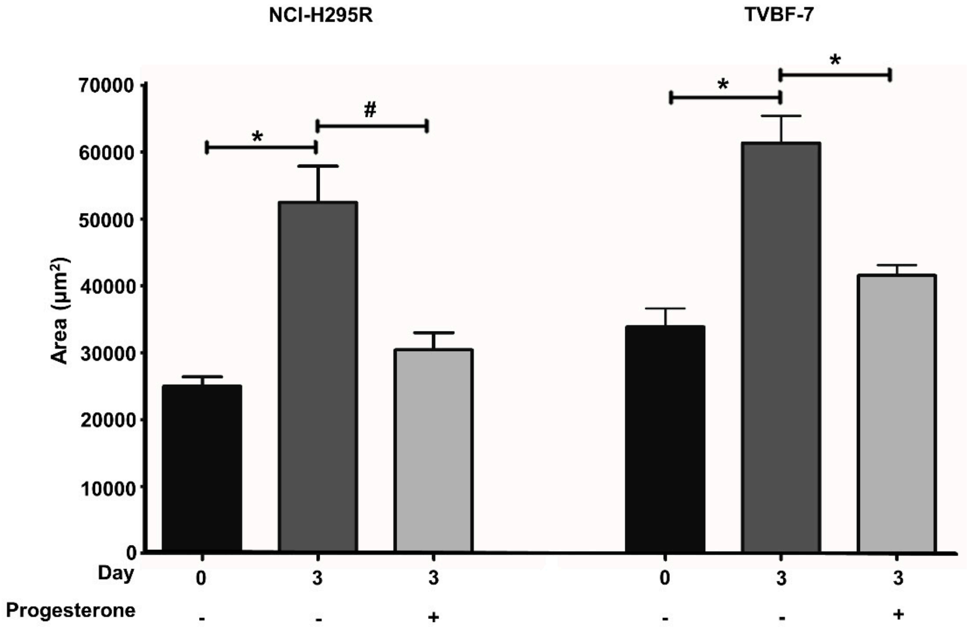

Supplementary Figure S2. Pg induced a reduction of the tumor xenograft area of NCI-H295R and TVBF-7 cells. Tumor areas of 48 hpf (T0 – the start of treatment) and 120 hpf (T3 – end of treatment) of vehicle-treated and 12,5 μM Pg-treated embryos xenografted with NCI-H295R or TVBF-7 cells. Data are shown as mean of independent experiments ± SEM. *$p < 0.0001$; # $p < 0.001$.

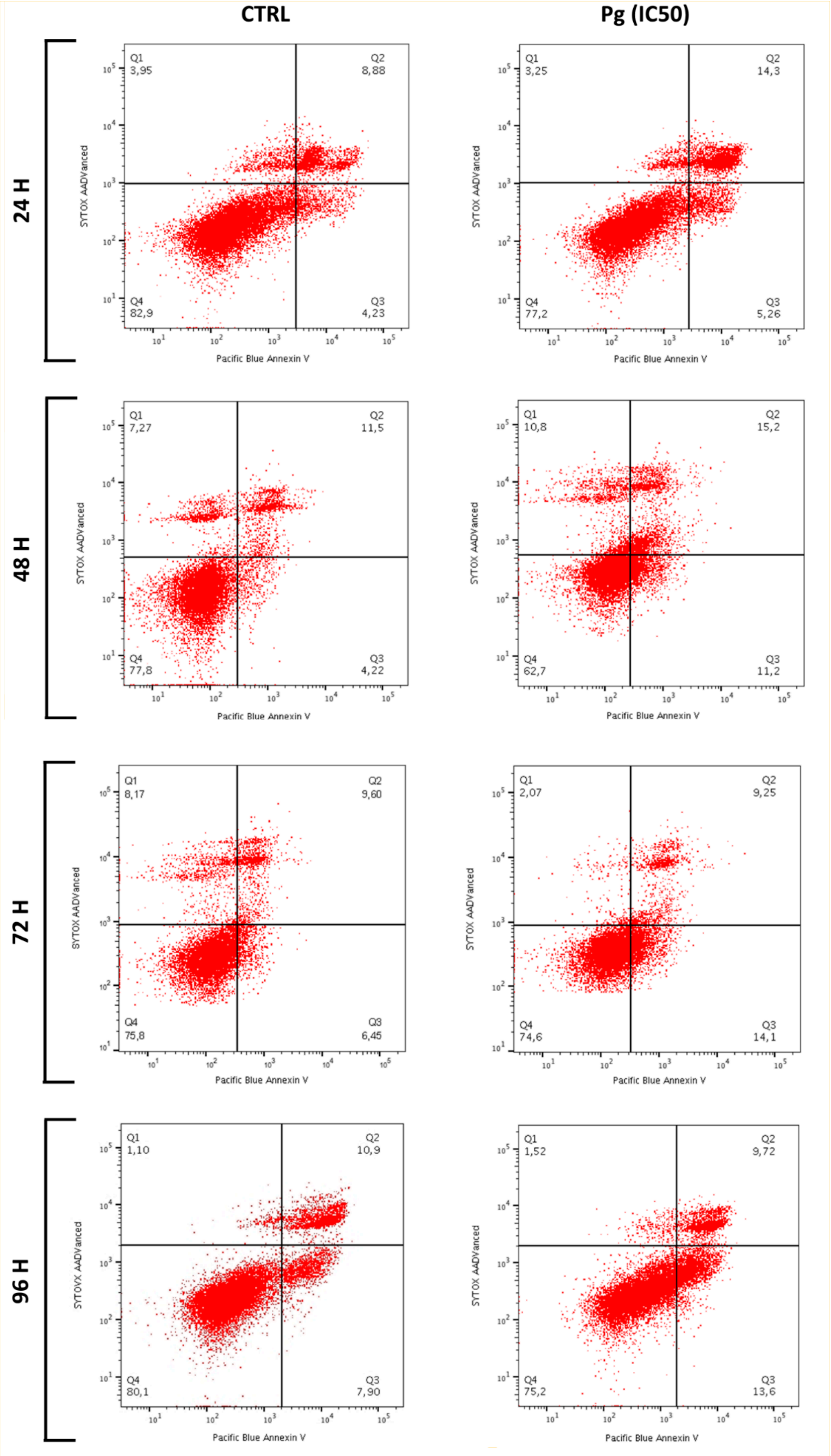

Supplementary Figure S3. Representative images of cell apoptosis in NCI-H295R cells after 24, 48, 72 and 96 hours of treatment with Pg. Alive cells are shown in the lower left part of the panel (Q4); early apoptotic cells are shown in the lower right part of the panel (Q3); late apoptotic cells

are shown in the higher right part of the panel (Q2); necrotic cells are shown in the higher left part of the panel(Q1).

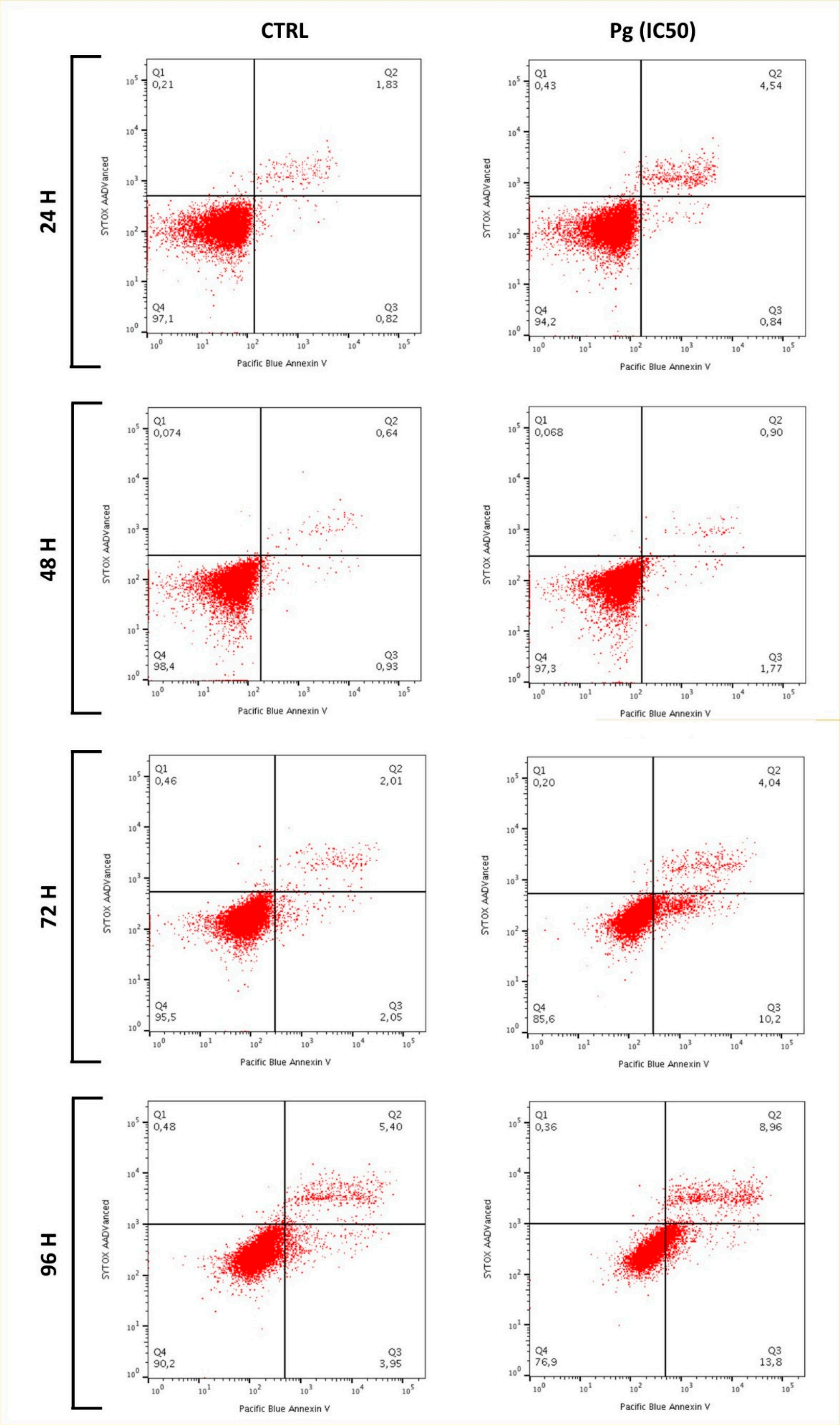

Supplementary Figure S4. Representative images of cell apoptosis in MUC-1 cells after 24, 48, 72 and 96 hours of treatment with Pg. Alive cells are shown in the lower left part of the panel (Q4); early apoptotic cells are shown in the lower right part of the panel (Q3); late apoptotic cells are

shown in the higher right part of the panel (Q2); necrotic cells are shown in the higher left part of the panel(Q1).

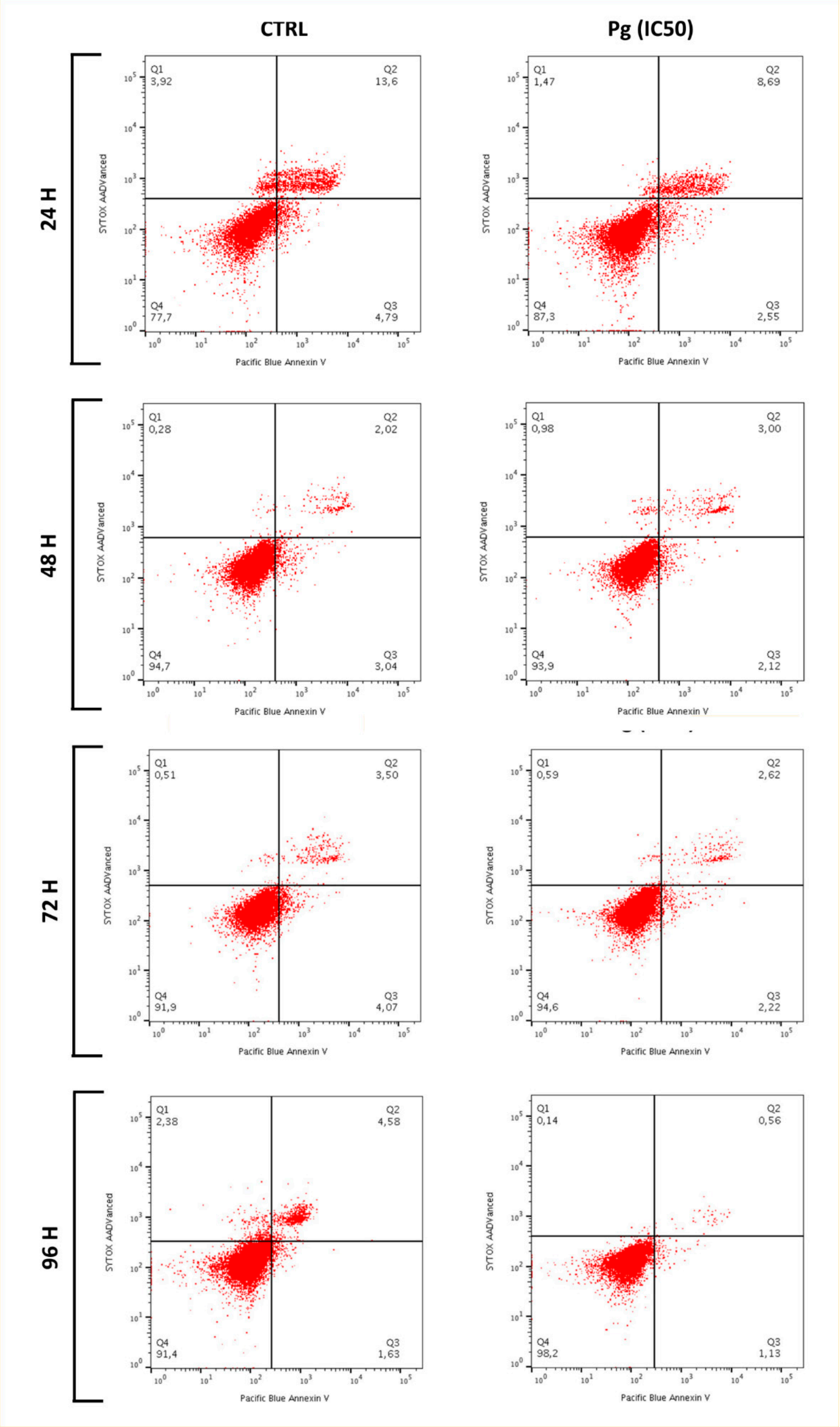

Supplementary Figure S5. Representative images of cell apoptosis in TVBF-7 cells after 24, 48, 72 and 96 hours of treatment with Pg. Alive cells are shown in the lower left part of the panel (Q4); early apoptotic cells are shown in the lower right part of the panel (Q3); late apoptotic cells are

shown in the higher right part of the panel (Q2); necrotic cells are shown in the higher left part of the panel(Q1).

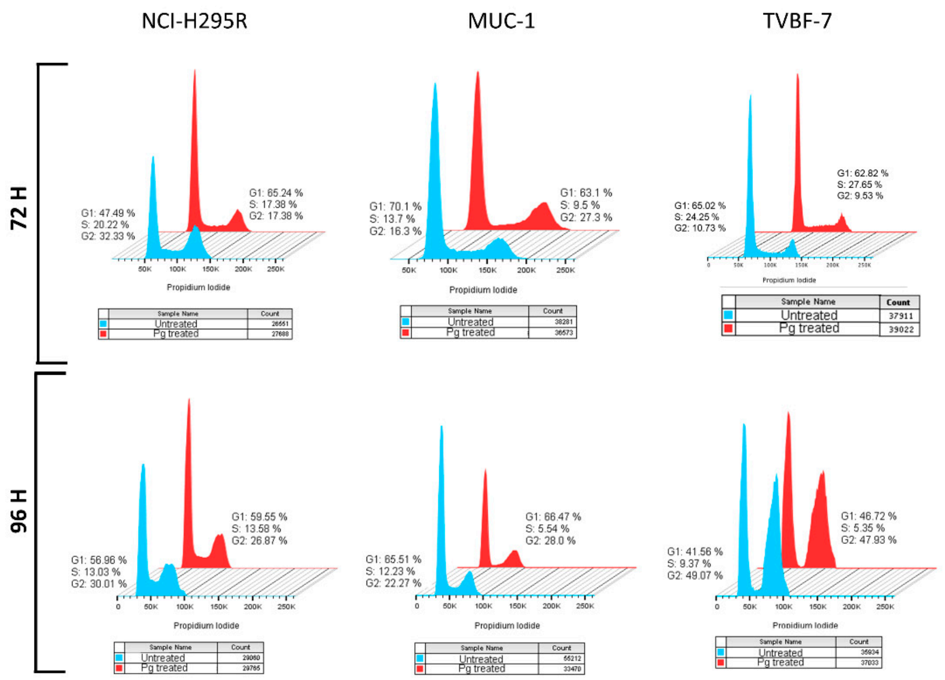

Supplementary Figure S6. Cell cycle analysis after Pg-treatment. Representative DNA histograms of NCI-H295R, MUC-1 and TVBF-7 untreated cells and 72h and 96h Pg-treated cells.

Supplementary Table S1. MMPs gene expression in ACC cell lines

	**NCI-H295R**	**MUC-1**	**TVBF-7**
MMP10	14,96	14,56	16,34
MMP11	11,70	10,12	15,08
MMP13	17,78	11,81	18,68
**MMP2**	**10,71**	**7,19**	**7,71**
MMP3	14,73	15,28	14,62
MMP7	19,48	12,06	Undetermined
MMP9	10,77	10,16	11,37

Values were reported as ΔCt, which are differences of the threshold cycle (Ct) values between the gene of interest and the β-actin housekeeping gene, calculated, as described in Materials and Methods.

Supplementary Table S2. Evaluation of the gene expression of MMP2 and its inhibitors in ACC cell lines.

	**Basal expression levels**			**Expression levels after Pg-treatment**		
**Target gene**	**NCI-H295R**	**MUC-1**	**TVBF-7**	**NCI-H295R**	**MUC-1**	**TVBF-7**
MMP2	11.01±0.48	7.74±0.1	7.47±0.1	11.14±0.17	8.41±0.07	7.55±0.05
TIMP1	5.98 ±0.32	7.74±0.09	6.80±0.22	5.59±0.17	8.41±0.08	6.80±0.28
TIMP2	5.44±0.28	2.52±0.16	9.24±0.06	4.77±0.22	2.35±0.14	8.95±0.03

Values were reported as ΔCt that are differences of the threshold cycle (Ct) values between the β-actin housekeeping gene and the gene of interest (ΔCt), calculated, as described in Materials and Methods.
